# Dispersion engineering of infrared epsilon-near-zero modes by strong coupling to optical cavities

**DOI:** 10.1515/nanoph-2023-0215

**Published:** 2023-06-21

**Authors:** Ben Johns

**Affiliations:** Department of Chemical Sciences, Indian Institute of Science Education and Research, Mohali, 140306, India

**Keywords:** dispersion engineering, epsilon-near-zero, Fabry–Perot cavity, phase change material, strong coupling

## Abstract

Epsilon-near-zero (ENZ) materials have recently emerged as a promising platform for infrared nanophotonics. A significant challenge in the design of ENZ-based optics is to control the dispersion of ENZ modes that otherwise have a flat profile near the ENZ frequency. Strong coupling with an optical cavity is a promising approach to ENZ dispersion engineering, which however has limitations due to the lack of tunability or nanofabrication demands of the cavity employed. Here, we theoretically and numerically show that much of the limitations of previous approaches can be overcome by strongly coupling the ENZ mode to an unpatterned Fabry–Perot cavity. We demonstrate this unprecedented ENZ dispersion control in coupled cavities by designing tunable infrared polarizers that can absorb *s* and reflect *p*-polarized components, or vice versa, for almost any oblique angle of incidence, i.e. omnidirectional polarizers. The feasibility of active control is also demonstrated using a phase change material within the cavity, which predicts dynamic switchability of polariton dispersions across multiple resonant levels at mid-infrared wavelengths. These results are expected to advance the current understanding of strongly coupled ENZ interactions and demonstrate their potential in tailoring dispersions for active and passive control of light.

## 1 Introduction

Coherent optical interactions lead to unusual phenomena such as Fano resonances [1], electromagnetically induced transparency [2], extraordinary optical transmission [3], surface lattice resonances [4], and strong coupling [5]. Strong coupling (SC) is characterized by a splitting of resonances when the frequencies of mutually coupled resonators or cavities are brought close to each other. This effect is typified by an anti-crossing of the cavity dispersions, which split to form two distinct (upper and lower) polariton branches [6]. The frequency separation at the anti-crossing point, known as Rabi splitting, signifies the strength of the mutual interaction, and SC can be experimentally observed in optical systems where the Rabi splitting is larger than the line-widths of individual resonances [7–12]. As a result of this splitting, SC opens up unique possibilities in engineering spectral response and dispersion in nanophotonics by finely tailoring polariton dispersions.

The introduction of SC has been proposed as an effective tool for exploiting the unique properties of epsilon-near-zero (ENZ) materials [13, 14]. ENZ materials have a vanishing or near-zero permittivity (*ϵ* → 0) at a particular wavelength and have attracted interest as a novel platform for exotic light–matter interactions [15]. Near the zero-epsilon wavelength (*λ*_*ZE*_), ENZ materials can exhibit extreme field concentration and enhancement, strong optical non-linearities and perfect absorption [16–21]. Moreover, the optical modes supported in ultrathin ENZ films, called ENZ modes [22], show strong field enhancements within low mode volumes, and have been utilized in active opto-electronic devices and ultrafast optical modulation [23–27]. Notably, the flat spectral dispersion near *λ*_*ZE*_ results in a zero group velocity, low propagation lengths, and it overall limits control over the operating frequencies or angles [28]. Earlier efforts in spectral shaping and tailoring of the ENZ mode dispersion explored its strong coupling with optical cavities such as quantum wells [14], gap plasmon modes [29], metasurfaces [30], phonon polaritons in polar dielectrics [31], plasmonic nanoantennas [32–34] and plasmon polaritons [28], which have helped realize negative refraction [13], hybrid plasmonic modes [28], and enhanced optical non-linearities [35]. However, the ability to tailor dispersions in strongly coupled systems is often hampered by the low tunability of the employed optical cavities. Moreover, the use of metasurfaces and plasmonic resonances involve nanofabrication processes that increase the cost and complexity of the system. Therefore, planar, lithography-free structures with large field enhancements and strong tunability need to be further explored as potential platforms to exploit strong coupling with ENZ modes.

Here, we overcome the limitations of previous strongly coupled ENZ designs and demonstrate unprecedented tailoring of polariton dispersions at infrared wavelengths by coupling to a Fabry–Perot (FP) cavity. FP cavities have long been the workhorse of strong coupling research, having been employed in polariton-enhanced transport [36], chemical reactivity [37], and condensation [38]; however, to the best of our knowledge, coupling of ENZ modes to a FP cavity has not been addressed earlier. Using an analytical approach, we identify the factors that control the coupling strength of the two modes, revealing that the polariton properties are mediated by an interplay of the near field of the ENZ and FP cavities as well as the ENZ thickness. In particular, we demonstrate the potential for ENZ dispersion engineering and complex spectral shaping in our system by designing planar, multilayer, coupled cavities that act as angle-independent, nearly omnidirectional polarizers with a controllable operation wavelength in the near and mid-infrared. Significantly, an analysis of the Hopfield coefficients of the strongly coupled cavities identifies the relative ENZ-like and cavity-like nature of the generated polaritons and highlights the possibilities in controlling the mixed nature of polaritons by tuning the FP cavity dispersion. Finally, to demonstrate their potential applications, active tunability of multilevel polariton dispersions is explored employing a phase change material. Our results not only demonstrate and characterize the extent to which ENZ mode dispersions can be engineered but also provide a simple configuration where ENZ light–matter interactions can be readily tailored for the versatile design of infrared optical components [39] and thermal emission control [40].

## 2 Results and discussion

### 2.1 Strong coupling of ENZ and FP modes

**Figure 1:**
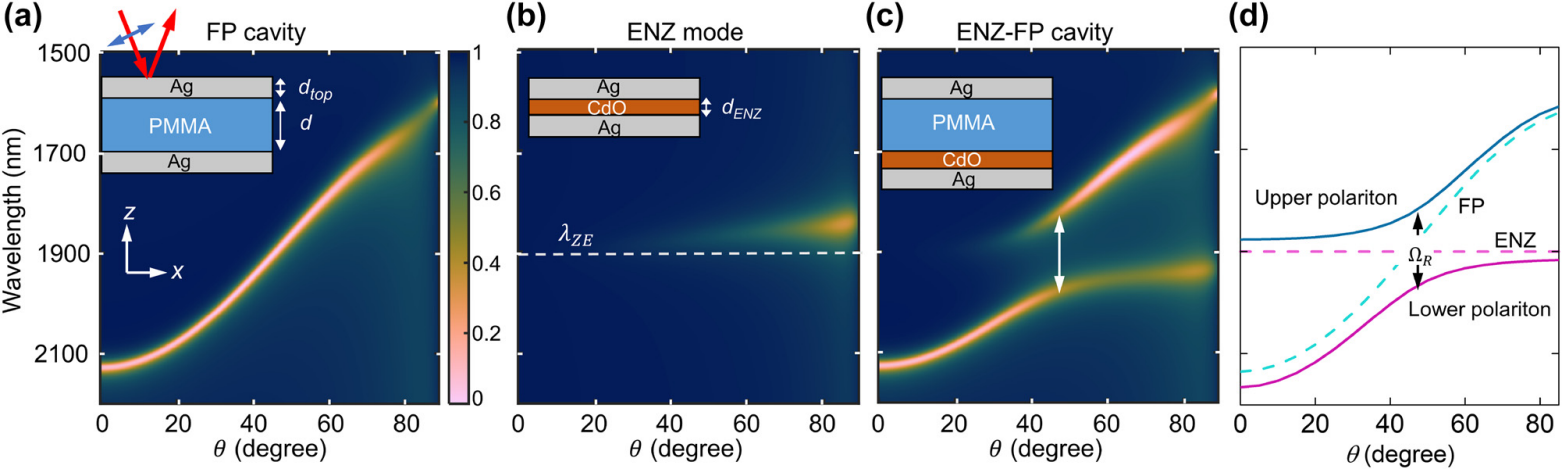
Strong coupling of ENZ and FP modes for *p*-polarized light. (a–c) Simulated reflectance color maps for *p*-polarized light incident on (a) FP cavity, (b) ENZ mode structure, and (c) ENZ-FP cavity. The corresponding geometries and coordinate axes are shown schematically as inset. The bottom Ag layer is semi-infinite while the top-most medium is assumed to be air in each case. The color scale is common in (a–c). (d) Calculated dispersion relations of the individual FP and ENZ modes (dashed curves) and the upper and lower branches of the strongly coupled dispersion (solid curves). The *y*-axis is common for (a–d). Arrows in (c, d) indicate the extent of splitting at the crossing point.

To investigate the possibility of strongly coupled resonances in a planar geometry, a structure composed of a dielectric (PMMA) and an ENZ layer (doped cadmium oxide, CdO) sandwiched between two metallic mirrors (Ag) is considered. The permittivity or refractive index data used in this work and their model parameters are given in section ‘Materials and Methods’ and plotted in Figure S1 in Supplementary Material. The individual or ‘bare’ resonances i.e. with only the dielectric between the mirrors (FP cavity: Ag-PMMA-Ag) and only the ENZ medium between the mirrors (ENZ mode: Ag–CdO–Ag) are first separately analyzed. The inset to Figure 1a schematically shows the FP structure, where the PMMA dielectric layer (thickness *d*) is sandwiched between the Ag substrate and a thin Ag layer of thickness *d*_top_. The thickness of the top metallic layer is comparable to its skin depth to allow coupling of light incident from the top-most air medium into the FP cavity. Throughout this work, the Ag substrate is assumed to be semi-infinite with transmittance *T* = 0. Figure 1a shows the calculated color map of reflectance (*R*) of the FP cavity for *p*-polarized light, i.e. *R*_*p*_, as a function of angle of incidence (*θ*) and wavelength (*λ*) in the near-IR (1500 nm–2200 nm). The thicknesses are set as *d* = 670 nm and *d*_top_ = 20 nm. The dispersion of the FP cavity is evident as a sharp dip in reflectance (bright region), and the wavelength of maximum absorption (*A* = 1 − *R*) varies over approximately a 500 nm range as *θ* is varied from 0 to 90° in Figure 1a. Figure 1b shows *R*_*p*_ for light incident from air onto the ENZ mode structure. Here, a CdO layer with a *λ*_*ZE*_ of 1900 nm and thickness *d*_*ENZ*_ = 20 nm is sandwiched between the Ag mirrors (see inset). The plot shows a flat dispersion in the vicinity of *λ*_*ZE*_, indicating the excitation of the radiative ENZ mode known as Berreman mode [22]. Importantly, the dispersions of the FP cavity and the ENZ mode can cross near *λ*_*ZE*_, leading to the possibility of strong coupling. Figure 1c shows *R*_*p*_ for the combined FP cavity-ENZ mode structure (Ag-PMMA-CdO-Ag, hereafter ENZ-FP cavity; see inset), revealing substantially modified absorption features. A clear anti-crossing of resonances is observed near *λ*_*ZE*_, characterized by a splitting of the overall dispersion into an upper and lower branch. This opens up a highly reflecting window around *λ*_*ZE*_ as indicated by the arrow in Figure 1c. The Rabi splitting (Ω_*R*_ = 55 meV) satisfies the criterion for strong coupling [6, 41], which is given as Ω_*R*_ > (*γ*_*FP*_ + *γ*_*ENZ*_)/2, where *γ*_*FP*_ ≈ 12 meV and *γ*_*ENZ*_ ≈ 25 meV are full widths at half maximum (FWHM) of the bare FP and ENZ resonances at the crossing point, respectively. The corresponding reflectance maps of the cavities for *s*-polarized light (*R*_*s*_) are shown in Figure S2, where the Berreman mode cannot be excited and only the FP resonance is observed in the ENZ-FP cavity. This clearly indicates that ENZ mode excitation is an integral part of the strongly coupled interaction observed in *R*_*p*_. Further, it is seen that the features in *R*_*s*_ may be effectively employed as a reference against which to compare the signatures of SC in *R*_*p*_.

To further understand the origin of the splitting, the frequency-wavenumber dispersion relations of the upper and lower branches are modeled by the following expression [28]:

(1) $$\begin{array}{ll} \omega^{\pm }\left(k\right) & =\frac{\omega_{FP}\left(k\right)+\omega_{\mathrm{ENZ}}\left(k\right)}{2}\\ & \;\pm \frac{1}{2}{\left[\Omega_{R}^{2}+{\left(\omega_{FP}\left(k\right)-\omega_{\mathrm{ENZ}}\left(k\right)\right)}^{2}\right]}^{1/2} \end{array}$$

where *ω* ^±^(*k*) denotes the dispersion relation of the upper (+) and lower (−) branches of the ENZ-FP cavity, *ω*_*FP*_(*k*) is the dispersion relation of the bare FP cavity, *ω*_*ENZ*_(*k*) is the dispersion relation of the bare ENZ mode, *k* = *k*_0_ sin*θ* is the in-plane wave number and *k*_0_ is the incident free-space wave number. The details of the calculation of *ω*_*FP*_(*k*) and *ω*_*ENZ*_(*k*) are given in Section S3. The strong coupling model in Equation (1) is presented in Figure 1d. *ω*_*FP*_(*k*) and *ω*_*ENZ*_(*k*) are plotted as dashed curves, while the upper and lower branches *ω* ^±^(*k*) are plotted as solid curves, calculated using Ω_*R*_ estimated from Figure 1c. For convenience, the point where the bare dispersions cross is referred to by (*ω*_*sc*_, *k*_*sc*_). The model qualitatively reproduces the observed anti-crossing behavior and splitting around this point well. It also shows how the upper and lower branches asymptotically tend to the bare dispersions away from (*ω*_*sc*_, *k*_*sc*_), validating the strongly coupled nature of interaction of the ENZ mode and the FP cavity at the crossing point. The anti-crossing behavior is further characterized in Supplementary Figure S4, which clearly shows mode splitting around *λ*_*ZE*_ and support the strongly coupled interaction picture in the ENZ wavelength regime.

### 2.2 Factors determining strong coupling

In this section, the factors affecting the strength of SC are systematically investigated. Strongly coupled systems have been classically described using a coupled harmonic oscillator model where the energy splitting depends on a coupling term in the coupled mode equations [6]. In optical systems, this coupling can be ascribed intuitively to the spatial overlap between electric fields of the interacting resonances at (*ω*_*sc*_, *k*_*sc*_) [10]. In the case of the ENZ-FP cavity, this can be written as [28]

(2) $$\Omega_{R}\propto \underset{V}{\int }E_{ENZ}\left(r\right).E_{FP}\left(r\right)\text{d}V$$

where **E**_*FP*_ is the electric field associated with the FP cavity, **E**_*ENZ*_ is the field of the ENZ mode, **r** is the position vector, the integral is over the cavity volume *V*, and the fields are evaluated at (*ω*_*sc*_, *k*_*sc*_). The ratio *g* of the frequency splitting Ω_*R*_ and the zero-epsilon frequency *ω*_*ZE*_,

(3) $$g=\frac{\Omega_{R}}{\omega_{ZE}}$$

can be used to further quantify the coupling strength between the ENZ mode and the FP cavity [42]. Several approximations can be made to simplify the analysis of Equation (2). First, the electric field of the ENZ mode is dominantly out-of-plane (along *z* direction, see coordinate axes in Figure 1a) [43]. Second, the out-of-plane ENZ mode fields in the ENZ-FP cavity are strongly confined to the interior of the ENZ layer, which results in the overlap integral being negligible outside the layer. Third, the field inside the ENZ layer is spatially uniform along *z* [43], allowing it to be taken outside the integral. These considerations allow Equation (2) to be simplified as

(4) $$\Omega_{R}\;\;\propto \;\;E_{z,ENZ}\overset{d_{\mathrm{ENZ}}}{\underset{0}{\int }}E_{z,FP}\left(z\right)dz$$

where the subscript ‘*z*’ denotes the out-of-plane component of the electric fields and the integral is now limited to the thickness of the ENZ layer.

**Figure 2:**
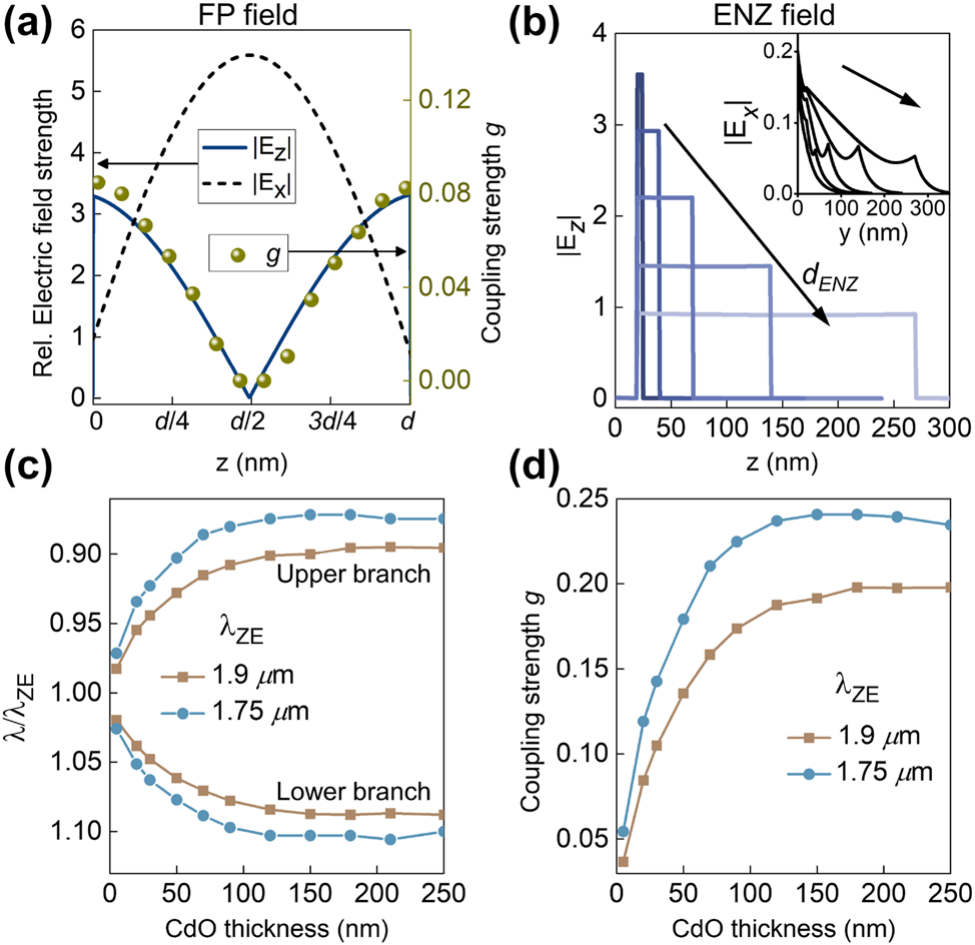
Dependence of coupling strength on electric field distribution and ENZ thickness. (a) Left axis: spatial variation of *E*_*z*,*FP*_ and *E*_*x*,*FP*_ field components (relative to the incident field strength) inside the bare FP cavity with vertical position (*z*) at (*ω*_*sc*_, *k*_*sc*_). Right axis: the dependence of the coupling strength *g* on the position of the ENZ layer relative to the FP cavity is co-plotted on the right axis. For this, *z* denotes the position of the center of the ENZ layer with respect to the FP cavity. (b) *E*_*z*,*ENZ*_ inside the bare ENZ cavity for *d*_*ENZ*_ varying from 5 to 250 nm at (*ω*_*sc*_, *k*_*sc*_). Corresponding *E*_*x*,*ENZ*_ plots are shown in the inset. Arrows indicate increasing *d*_*ENZ*_. (c) Resonance wavelength splitting of the ENZ-FP cavity at (*ω*_*sc*_, *k*_*sc*_) as a function of varying *d*_*ENZ*_ for *λ*_*ZE*_ = 1.9, 1.75 μm. (d) Corresponding variation in *g* with *d*_*ENZ*_.

Based on Equation (4), the dependence of coupling strength on the FP cavity field is investigated first. Figure 2a (left axis) plots the components of electric field as a function of vertical position inside the bare FP cavity. The fields are plotted for the FP cavity in Figure 1 (*d* = 670 nm, *d*_top_ = 20 nm), at the point (*ω*_*sc*_, *k*_*sc*_). The plot shows that while the in-plane component (*E*_*x*,*FP*_) is minimum at the surface of the Ag mirrors, the out-of-plane component (*E*_*z*,*FP*_) is maximum as a consequence of the Fresnel reflection phase imparted by the perfect electric conductor-like metal. Equation (4) suggests that the strong coupling interaction, which is expected to be mediated by the *z* components, will be maximized when the location of the ENZ layer is at the bottom (or top) of the ENZ-FP cavity (where *E*_*z*,*FP*_ is maximum) and minimized when the ENZ layer is at the center of the cavity. To verify this, the coupling strength *g* for the ENZ-FP cavity in Figure 1 is calculated as a function of the vertical position of the ENZ layer in the cavity and is plotted on the right axis of Figure 2a. Here, the ENZ thickness is a constant and only its position within the cavity is varied. It is evident that *g* is maximum when the ENZ layer is at the surface of the Ag mirrors and is zero when it is placed at the center of the cavity, in close correlation with the strength of *E*_*z*_ of the FP cavity. Note that this is in contrast to most observations in literature where the strong coupling is maximum when the active layers with dominantly in-plane fields or dipole moments are placed at the center of the FP cavity [44].

Having identified the role of the out-of-plane FP field strength, the dependence of Ω_*R*_ on the ENZ parameters i.e., *d*_*ENZ*_ and *E*_*ENZ*_ are considered next. To characterize this, the wavelength splitting of the upper and lower branches of the ENZ-FP cavity are calculated for different ENZ thicknesses. Figure S5 plots *R*_*p*_ of the ENZ-FP cavity in Figure 1 for different ENZ thicknesses from 0 to 250 nm. From this, the wavelength splitting is calculated after identifying the respective cross-over points of the bare dispersions for each thickness. Figure 2c plots the upper and lower polariton wavelengths (normalized to *λ*_*ZE*_) against *d*_*ENZ*_, calculated for *λ*_*ZE*_ = 1750 and 1900 nm. The splitting is observed to initially increase with ENZ thickness but saturates around *d*_*ENZ*_ ∼ 100 nm for both values of *λ*_*ZE*_. Figure 2d further plots the variation of *g* with *d*_*ENZ*_, clearly showing the initial sharp increase in the coupling strength and its saturation at larger values of ENZ thickness for both the values of *λ*_*ZE*_. To understand this, two cases are analyzed here: I. *d*_*ENZ*_ is a small fraction of the overall cavity thickness (*d*_*ENZ*_ ≪ *d*) and II. *d*_*ENZ*_ ∼ *d*. In case I, Equation (4) can be further simplified by assuming that the spatial variation in *E*_*FP*_ is negligible over the scale of the ENZ layer thickness, bringing it outside the integral. This yields Ω_*R*_ ∝ *E*_*z*,*FP*_ *E*_*z*,*ENZ*_ *d*_*ENZ*_, which indicates that Ω_*R*_ increases in proportion to *d*_*ENZ*_ when the ENZ thickness is low enough. More accurately, Ω_*R*_ is decided by the inter-relation between *d*_*ENZ*_ and its mode field, *E*_*z*,*ENZ*_. Figure 2b plots *E*_*z*_ inside the bare ENZ cavity when *d*_*ENZ*_ is varied from 5 nm to 250 nm, at (*ω*_*sc*_, *k*_*sc*_) where it crosses the FP dispersion of Figure 1. The *E*_*z*_ component is seen to be spatially uniform even for the thickest 250 nm CdO layer. The corresponding plots of *E*_*x*,*ENZ*_ are shown in the inset, which are an order of magnitude weaker than *E*_*z*,*ENZ*_, validating the assumptions stated earlier that the field in the ENZ mode is dominantly out-of-plane and spatially uniform in nature. Notably, *E*_*z*,*ENZ*_ decreases as *d*_*ENZ*_ increases, which means that the expected increase in coupling strength with *d*_*ENZ*_ due to a larger interacting volume would be tempered by the decreasing ENZ mode field strength. Thus, the coupling strength will be determined by a trade-off between the larger interaction volume at large *d*_*ENZ*_ and the stronger interacting electric field at small *d*_*ENZ*_ in the ENZ layer.

The initial increase in coupling strength in Figure 2d follows from the expected dependence of Ω_*R*_ on *d*_*ENZ*_ (larger interaction volume) discussed in case I. The increase is however sub-linear, pointing to the effect of the decreasing *E*_*z*,*ENZ*_ that reduces the interaction strength. This does not, however, explain the saturation of *g* at large *d*_*ENZ*_, for which case II (*d*_*ENZ*_ ∼ *d*) is considered. Here, it is evident that the variation in *E*_*FP*_ over the scale of the ENZ layer thickness is no longer negligible. In fact, increasing the ENZ thickness can be imagined to be similar to adding ENZ layers sequentially towards the center of the FP cavity. As discussed earlier, this would contribute negligibly to the overall coupling strength due to the decay of *E*_*z*,*FP*_ towards the center. Therefore, effectively only the ENZ layers near the surface of the Ag mirrors will be involved in SC, which qualitatively explains why *g* saturates at large values of *d*_*ENZ*_.

### 2.3 Wide-angle polarizer design

**Figure 3:**
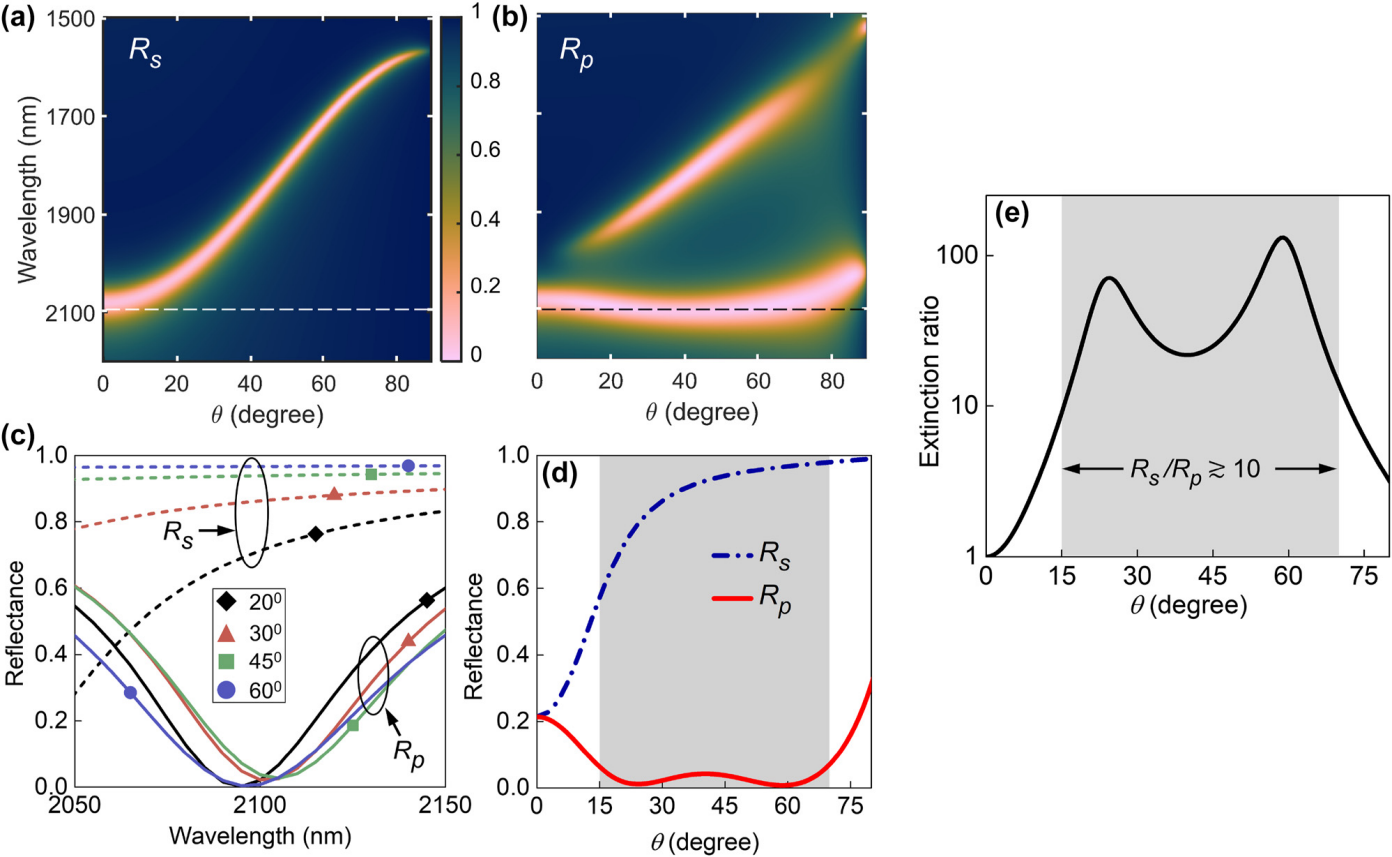
Design of wide angle reflective *s*-polarizer. (a, b) Calculated reflectance maps (a) *R*_*s*_ and (b) *R*_*p*_ of the Ag-PMMA-CdO-Ag cavity optimized as a wide-angle reflective *s*-polarizer. The target wavelength *λ* = 2100 nm is indicated by the horizontal lines. (c) Spectral variation of *R*_*s*_ and *R*_*p*_ at different incident angles around *λ* = 2100 nm. (d) Angle-dependence of *R*_*s*_ and *R*_*p*_ plotted at *λ* = 2100 nm. (e) *R*_*s*_/*R*_*p*_ at *λ* = 2100 nm showing a reflectance contrast > 10 for angles between 15 and 70°.

Having identified the control parameters for strong coupling in an ENZ-FP cavity, in this section, the design of infrared wide-angle polarizers is demonstrated to highlight the extent of dispersion engineering possible in this system. Figure 3a and b shows the reflectance maps *R*_*s*_ and *R*_*p*_ of an Ag-PMMA-CdO-Ag cavity designed to act as a wide-angle, reflective *s*-polarizer at a target near-IR wavelength *λ* = 2100 nm. The parameters of the cavity are *d* = 522 nm, *d*_top_ = 9 nm, *d*_*ENZ*_ = 128 nm and *λ*_*ZE*_ = 1.985 μm, as summarized in Table 1. The numerical optimization process is outlined in Section S6. In Figure 3a, although *R*_*s*_ is very low for near-normal incidence at *λ* = 2100 nm, the strong angle-dependence of the bare FP dispersion ensures that the cavity strongly reflects *s*-polarized light at oblique angles ≳15°. On the other hand, at this wavelength the cavity shows strong absorption of *p*-polarized light for almost any angle of incidence (Figure 3b), resulting in an extremely low *R*_*p*_ over a wide range of *θ*. This is achieved by engineering the SC in the ENZ-FP cavity so that its lower polariton branch lies near *λ* = 2100 nm and possess an extremely flat dispersion, as evident in Figure 3b. This large contrast between *R*_*s*_ and *R*_*p*_ over a wide range of oblique angles effectively makes the ENZ-FP cavity a nearly omnidirectional *s*-polarizer in reflection mode. To quantify the performance of the polarizer at and around its target wavelength, Figure 3c shows the contrast between *R*_*s*_ and *R*_*p*_ around *λ* = 2100 nm for four angles in the range 20–60°. The minima of *R*_*p*_ lie consistently close to zero near 2100 nm and show negligible spectral variation with *θ*, while *R*_*s*_ remains high throughout, showing the large and nearly angle-independent nature of the reflectance contrast. To further verify the extent of omnidirectionality, Figure 3d plots the reflectance as a function of *θ* at 2100 nm. Away from normal incidence, *R*_*s*_ steadily increases while *R*_*p*_ decreases to nearly zero. In the shaded region where *θ* lies between 15° and 70°, *R*_*s*_ varies from ≈60 % to 98 % while *R*_*p*_ lies below 7 % throughout this range, with an average value of 3 %. The extinction ratio, defined as the ratio of the power in orthogonal polarizations [39] (here *R*_*s*_/*R*_*p*_) is plotted in Figure 3e. In the range *θ* = 15°–70°, the extinction ratio is at least 10 and goes up to as high as 10^2^, showing that the designed ENZ-FP cavity can function as a polarizer with a wide working range of incident angles and high efficiency.

**Table 1:** Summary of numerically optimized parameters for wide-angle polarizers.

Parameter	*s*-polarizer	*p*-polarizer
Target wavelength	2100 nm	4000 nm
Top metallic layer	Ag, *d*_top_ = 9 nm	CdO, *d*_top_ = 120 nm
Dielectric layer	PMMA, *d* = 522 nm	GST, *d* = 107 nm
CdO ENZ layer	*d*_*ENZ*_ = 128 nm, *λ*_*ZE*_ = 1.985 μm	*d*_*ENZ*_ = 118 nm, *λ*_*ZE*_ = 3.98 μm

**Figure 4:**
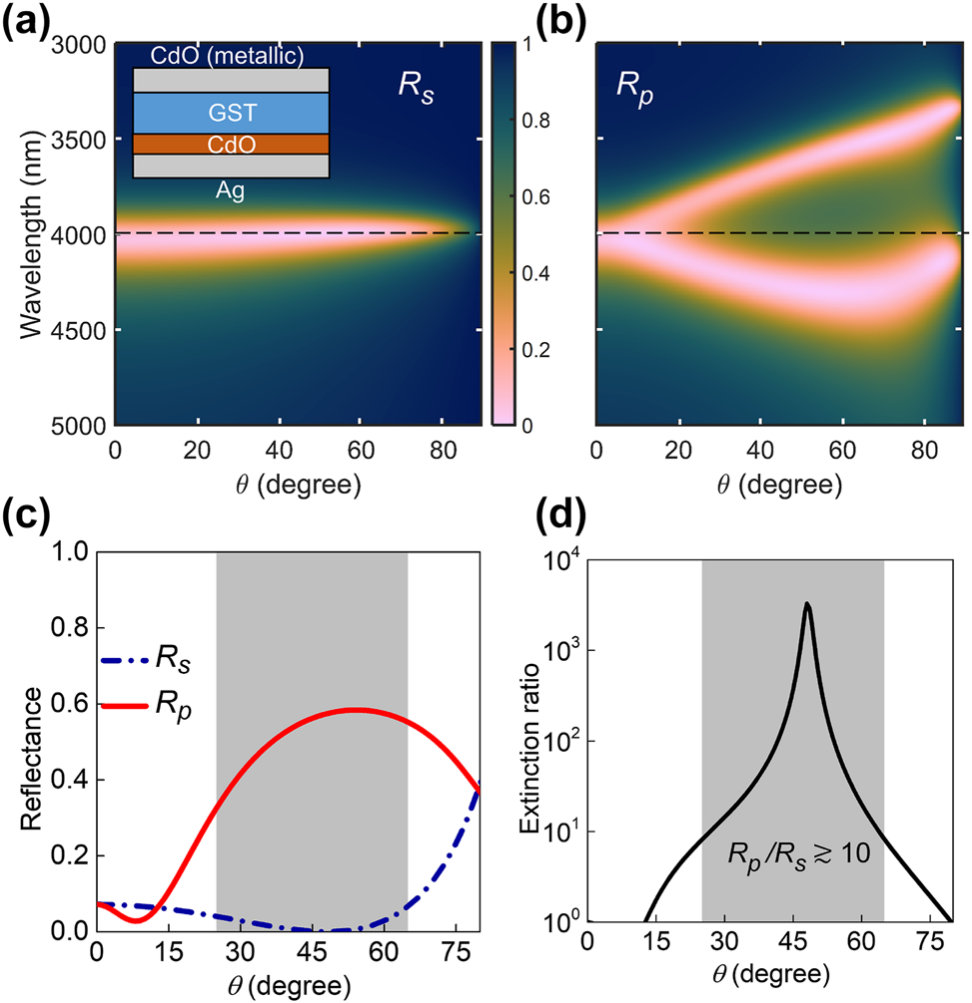
Design of wide angle reflective *p*-polarizer. (a, b) Calculated reflectance maps (a) *R*_*s*_ and (b) *R*_*p*_ of the mid-IR ENZ-FP cavity, shown schematically in the inset. The target wavelength *λ* = 4000 nm is indicated by the horizontal lines. (c) Angle-dependence of *R*_*s*_ and *R*_*p*_ plotted at *λ* = 4000 nm. (d) *R*_*p*_/*R*_*s*_ at *λ* = 4000 nm showing an extinction ratio ∼10 to 10^3^ for angles between 25 and 65°.

Following this, a reflective *p*-polarizer with a wide angular range is also realized based on SC in the ENZ-FP cavity. Such a device would have omnidirectional absorption of *s*-polarized light and high reflectance for *p*-polarized light, requiring opposite results to that in Figure 3. Polarizer schemes based on thin film polaritonic absorbers usually involve absorption of the *p*-component since surface polaritons are excited only by transverse magnetic fields [22]. In light of this, a polarizer that can reflect *p*- and absorb *s*-polarized light over wide angles would be an important advancement. This requires an all-angle absorber for *s*-polarization with a very flat dispersion at the target wavelength. However, *R*_*s*_, which is determined by the bare FP cavity resonance, shows a prominently dispersive behavior. For example, in Figure 3a the resonance wavelength varies by ∼500 nm in the near-IR as *θ* is varied from 0 to 90°. To overcome this dispersive nature of the FP resonance and obtain angle-independent absorption, crystalline germanium antimony telluride (GST) having an extremely high refractive index (*n* > 6) with a simultaneously low loss (*k* ≈ 0.05) in the mid-IR window from 3 to 5 μm [45] is used as the cavity dielectric. Section S7 further discusses the dependence of the FP resonance on the dielectric refractive index. Due to operation in the mid-IR window (3–5 μm), the Ag top layer is replaced with a metallic CdO layer with *λ*_*ZE*_ = 2.1 μm. The metallic CdO layer only plays the role of mirror in the FP cavity, which is used because Ag becomes too reflective to allow light to pass into the cavity. Setting a target operation wavelength of *λ* = 4 μm for the polarizer, Figure 4a shows *R*_*s*_ for an ENZ-FP cavity (shown schematically in the inset), revealing an extremely flat dispersion independent of *θ* at the target wavelength. The numerically optimized parameters for this ENZ-FP cavity are *d*_top_ = 120 nm, *d* = 107 nm, *d*_*enz*_ = 118 nm and *λ*_*ZE*_ = 3.98 μm (Table 1). The corresponding *R*_*p*_ map is shown in Figure 4b, showing the upper and lower polaritonic branches of the ENZ-FP cavity. The strong coupling ensures a large splitting between the upper and lower branches at oblique incidence, which opens up a reflecting spectral window in *R*_*p*_ around the target wavelength over a wide angular range (Figure 4b). It is interesting to note that the numerically optimized ENZ wavelength of *λ*_*ZE*_ = 3.98 μm is spectrally coincident with the FP resonance in Figure 4a, which facilitates the opening of the reflecting window at the target wavelength. To demonstrate the wide angular range of the reflectance contrast, Figure 4c plots *R*_*s*_ and *R*_*p*_ at *λ* = 4 μm as a function of *θ*. A large contrast in reflectance at angles between 25° and 65° in the shaded region is evident where *R*_*p*_ varies from ≈35 % to 60 % while *R*_*s*_ lies below 7 % throughout this range, with an average value of 2 %. Further, the extinction ratio (here *R*_*p*_/*R*_*s*_) plotted in Figure 4d demonstrates that the ratio is greater than 10 within the shaded region between 25° and 65°, reaching a maximum value > 3000 around 50° where *R*_*s*_ goes to zero. Thus, engineering the ENZ-FP dispersion via SC is shown to give a high-efficiency, wide-angle *s-* polarizer in the mid-IR.

### 2.4 Analysis of Hopfield coefficients of strongly coupled ENZ-FP modes

**Figure 5:**
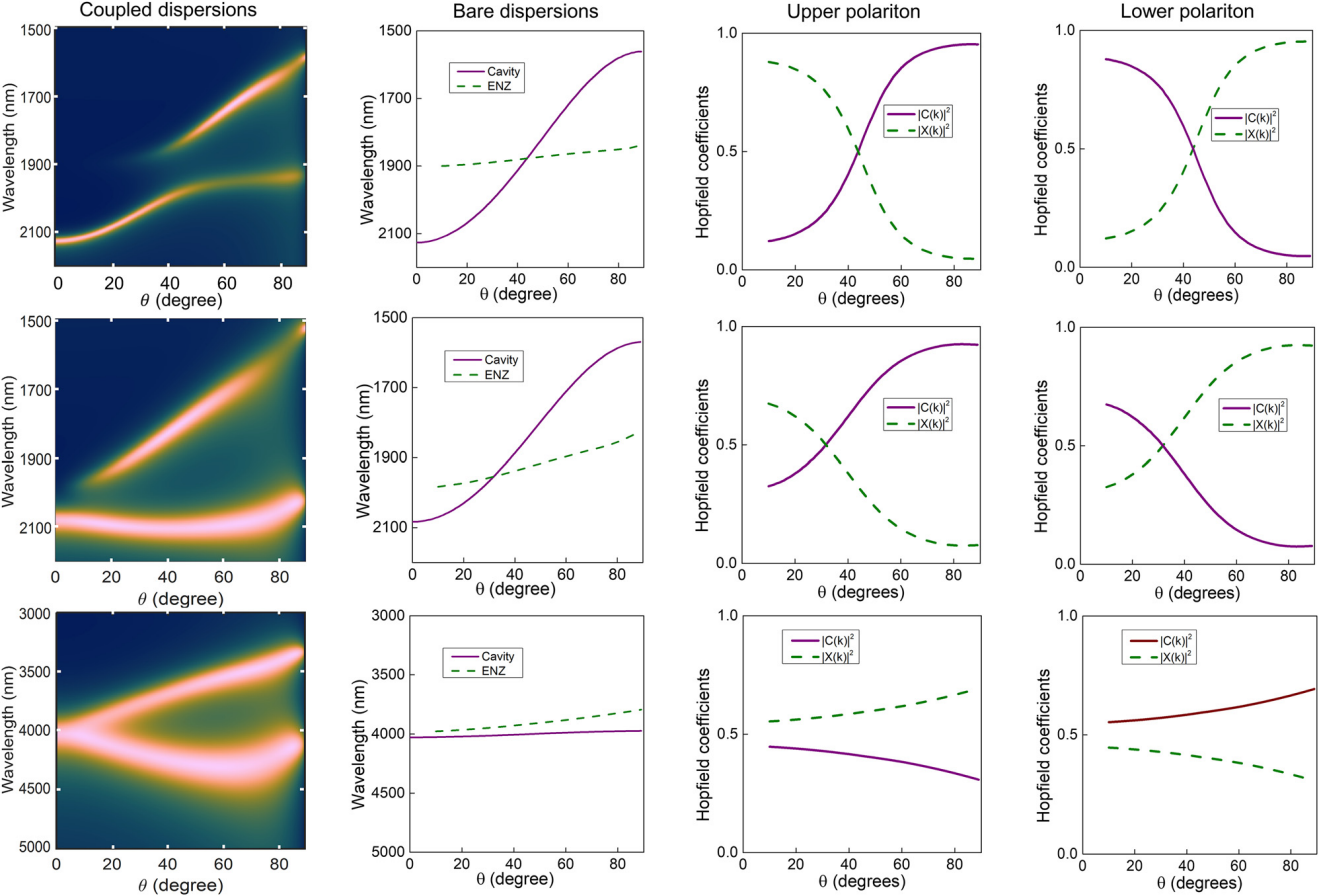
Analysis of Hopfield coefficients. Column 1 shows *R*_*p*_ maps of the three cavities presented in Figure 1, Figure 3, and Figure 4. Column 2 shows the corresponding bare dispersion relations of the FP and ENZ cavities. The last two columns plot the Hopfield coefficients for the upper and lower polaritons, respectively. Here, *X*(*k*_‖_) and *C*(*k*_‖_) represents ENZ and FP fraction, respectively.

From a more fundamental perspective, an interesting question that arises is on the extent to which the generated polaritons inherit the ENZ or cavity nature of the Berreman mode and the FP cavity. As the hybridized polaritons are known to partially inherit properties of both interacting modes, it becomes possible to realize otherwise unattainable mode properties. For example, it was shown that the field confinement and propagating nature of a non-radiative ENZ mode can be controlled by hybridizing it with a surface plasmon polariton (SPP) [28]. A similar analysis has shown that the effective field strengths of the generated polaritons are determined by the relative contributions (fractions) of ENZ and SPP modes [31]. In another approach towards (effective) ENZ polaritons using metal-dielectric-metal cavities, it has been shown that polaritons generated from these cavities inherit their ENZ nature [46]. To gain insight into the ENZ/cavity mixed nature of the polaritons in the ENZ-FP cavity here, an analysis of Hopfield coefficients of the coupled modes in Figure 1, Figure 3, and Figure 4 is performed. In the quantum mechanical picture of strong coupling, Hopfield coefficients (as a function of the in-plane wave number *k*) indicate the relative contribution of the FP cavity state (*C*(*k*)) and the ENZ state (*X*(*k*)) to the upper and lower polariton wave functions such that they obey the relation |*C*(*k*)|^2^ + |*X*(*k*)|^2^ = 1. The Hopfield coefficients for the upper polariton are given by [47]

(5a) $$|C\left(k\right){|}^{2}=\frac{1}{2}\left(1-\frac{\Delta E\left(k\right)}{\sqrt{\left(\Delta E{\left(k\right)}^{2}+\Omega_{R}^{2}\right)}}\right)$$

(5b) $$|X\left(k\right){|}^{2}=\frac{1}{2}\left(1+\frac{\Delta E\left(k\right)}{\sqrt{\left(\Delta E{\left(k\right)}^{2}+\Omega_{R}^{2}\right)}}\right)$$

where Δ*E*(*k*) is *ℏ*(*ω*_*FP*_(*k*) − *ω*_*ENZ*_(*k*)), and the values of |*C*(*k*)| and |*X*(*k*)| are exchanged for the lower polariton. While they have been mainly used in analyzing cavity QED systems (e.g., calculating polariton effective mass [44]), Hopfield coefficients are also useful in purely optical systems to gain insight into the intermixing of different optical modes [31]. Figure 5 shows the coupled and bare dispersions as well as the Hopfield coefficients for three strongly coupled cavities. In row 1, a typical strongly coupled ENZ-FP system (from Figure 1) is shown, whose Hopfield coefficients show that the relative ENZ and cavity nature of the generated polaritons possess an expected strong angle (or *k*) dependence. At low angles, the upper polariton (UP) is dominantly ENZ-like (|*X*(*k*)|^2^ > 0.5). As *θ* increases, the bare dispersions approach each other and at the crossing point, |*C*(*k*)|^2^ = |*X*(*k*)|^2^, signifying equal ENZ and FP cavity nature. At higher angles, the UP becomes increasingly FP cavity-like (|*C*(*k*)|^2^ > 0.5), as also evident from the its coupled dispersion plot in the first column. The lower polariton (LP) shows the opposite trend, with a dominant cavity-like behavior at low angles, before approaching the ENZ wavelength, and finally takes on an ENZ mode-like behavior at large *θ*. Row 2 shows the coupled and bare dispersions as well as the Hopfield coefficients of the *s*-polarizer cavity (from Figure 3). As seen from the bare dispersions, the FP and ENZ modes cross at a lower angle than in row 1, hence the UP changes from ENZ-like to cavity-like also at a lower angle. Similarly, LP shows crossover from cavity-like to ENZ-like at this angle. The fact that only the LP is excited at normal incidence in rows 1 and 2 further shows that the UP in these coupled dispersions are ENZ-like at low angles, since the ENZ mode will not be excited at normal incidence.

The behavior found in row 1 and 2 is consistent with strongly coupled systems comprising a dispersive and a non-dispersive resonance (e.g., a FP cavity and semiconductor exciton [5] or an SPP and ENZ mode [28]), wherein the polaritons contain equal fractions of the interacting modes at the cross-over point, while away from the cross-over they tend to have a dominant contribution from one of the interacting modes [36–38]. Interestingly, the Hopfield coefficients in the bottom row of Figure 5 corresponding to the *p*-polarizer cavity (from Figure 4) deviate significantly from this trend. Here, a novel strong coupling interaction occurs where *both* the bare modes are nearly non-dispersive with the FP and ENZ modes having a flat profile near the ENZ wavelength. As a result, the Hopfield coefficients also show a suppressed angle-dependence and both UP and LP possess substantial (∼0.5) ENZ fraction at almost any angle of incidence. In other words, both polaritons show a significant mixed nature *irrespective of the proximity to the crossing point.* This is in marked contrast to conventional strong coupling interactions between two resonances where a significant mixed nature is obtained only in a small range of wave numbers (and energies) around the crossing point. Such a behavior raises the exciting prospect of realizing hybrid polaritonic properties over a much larger range of wave numbers. Moreover, the UP, which is more ENZ-like, acquires considerable dispersive nature compared to the bare ENZ mode (due to its substantial cavity fraction). It is also noteworthy that the LP, which has a slightly greater cavity fraction than ENZ fraction, shows a negative group velocity at low-k values, a feature that has also been seen in high-k modes of strongly coupled ENZ dispersions [31]. To summarize, the Hopfield coefficients for the three cavities clearly evidence the mixed nature of the generated polaritons and reveals that the relative ENZ/FP nature can be finely controlled (around and even well beyond the crossing point) by rational design of the bare ENZ and FP dispersions in our simple, planar multilayer structure.

It is also worth mentioning here that these cavities also hold promise in thermal photonics applications. For example, Figure 4a shows an omnidirectional, wavelength-selective perfect absorber, which is relevant for thermal imaging applications in the 3–5 μm atmospheric transparency window [48]. Moreover, both the structures shown in Figures 3 and 4 are highly polarized, wavelength-selective thermal emitters, according to Kirchhoff’s law [49]. Significantly, previous demonstrations of wide-angle, selective emitters have utilized photonic crystals and nanostructures, which present a much larger scale of fabrication complexity compared to the multilayer structures here [50]. Such spectrally-selective, quasi-monochromatic thermal emission is a sought-after feature in thermophotovoltaic (TPV) energy conversion technologies [49, 51]. However, it should be noted that the devices need to be heated to elevated temperatures to radiate at their characteristic wavelengths (1380 K and 725 K corresponding to black body radiation peaking at 2.1 μm and 4 μm, respectively). This could trigger temperature-dependent variations in the material properties, e.g., GST’s phase transition can be induced by heating around 430 K [48] and the free carrier density in doped CdO may get quenched at higher temperatures [21]. Therefore, any such changes in the optical response of the materials with temperature also have to be taken into account to accurately predict thermal emission of these cavities at higher temperatures. Another attractive feature of these cavities is their tunability. Although the polarizers are designed here at only a particular infrared wavelength, there is nothing special about the demonstrated operation wavelengths of *λ* = 2100 nm and 4000 nm. To illustrate this, note that in addition to the geometric parameters of the cavity, the dielectric parameters of all the materials employed here are also highly tunable. For instance, the ENZ wavelength of transparent conducting oxides such as ITO and doped CdO can be tuned over a wide range of wavelengths [21, 28]. This critical property is what allows *λ*_*ZE*_ to be included as an optimization parameter in our calculations. Furthermore, GST is a phase change material showing non-volatile switching of its optical response in the visible and infrared regions [52–54]. Thus, apart from the static tunability, this opens up the exciting possibility of dynamically tuning the strongly coupled cavity interactions [49].

### 2.5 Dynamic multi-level resonances in ENZ-FP cavity

**Figure 6:**
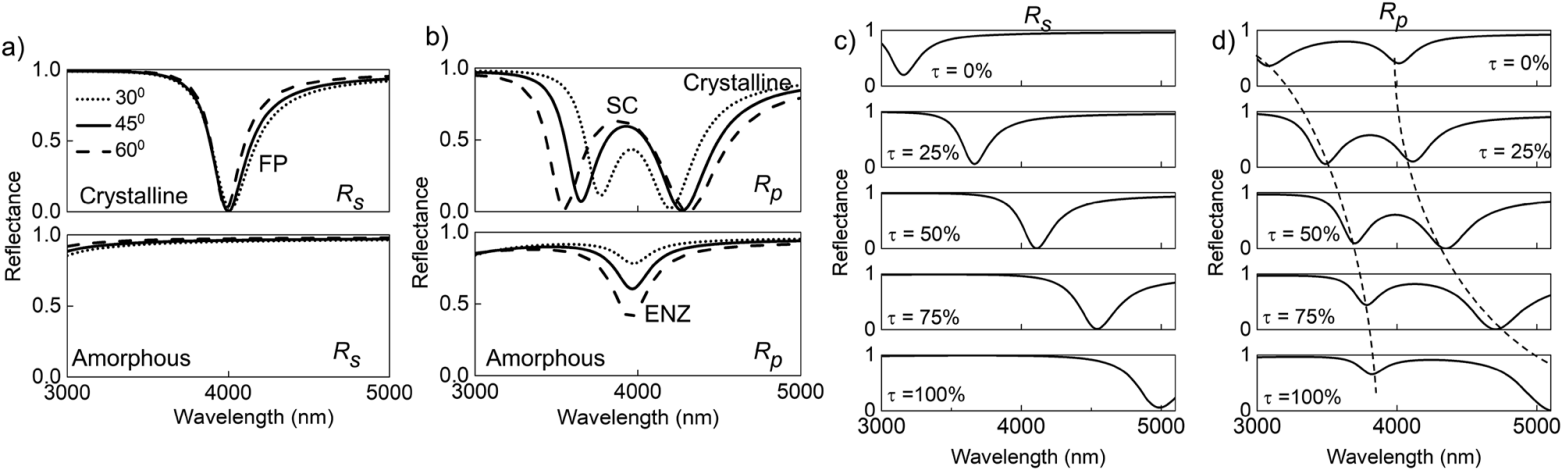
Dynamic tuning of strong coupling in ENZ-FP cavity. (a) Wavelength dependence of *R*_*s*_ of the mid-IR ENZ-FP cavity for crystalline GST (top) and amorphous GST (bottom). The dip in *R*_*s*_ is due to the FP resonance. (b) Corresponding plots of *R*_*p*_ for the ENZ-FP cavity. The top panel shows strong coupling (SC) while the dip in the bottom panel is due to the ENZ mode. The legend showing angle of incidence is common to (a) and (b). Reflectance spectra (c) *R*_*s*_ and (d) *R*_*p*_ of the mid-IR ENZ-FP cavity at an angle of incidence 45° for different values of the crystallization fraction (*τ*) of GST. Dashed lines in (d) indicate the avoided crossing of resonances.

In this section, the potential for active tunability of the ENZ-FP cavity by exploiting phase change in GST is explored. Figure 6a compares the wavelength dependence of *R*_*s*_ of the mid-IR ENZ-FP cavity for three values of *θ* when GST is in its crystalline phase (top panel) and amorphous phase (bottom). A remarkable variation in *R*_*s*_ is evident with the cavity changing from a nearly omnidirectional perfect absorber at *λ* = 4 μm to a perfect reflector of *s*-polarized light on switching the phase of GST. The underlying reason here is that the FP resonance at 4 μm for crystalline GST has shifted to much lower wavelengths due to the lower refractive index of GST in its amorphous phase (*n* ≈ 3.5) [45]. Figure 6b shows the corresponding plots of *R*_*p*_. Here, the strongly coupled resonances in the crystalline phase are evident as dual reflectance dips in the top panel. However, the dual resonances disappear when GST changes to its amorphous phase leaving a single resonance at the ENZ wavelength of CdO. This is because the FP resonance in the amorphous phase is spectrally separated from the ENZ mode, and the only response of the ENZ-FP cavity for *p*-polarized light in the 3–5 μm range is the absorption of the ENZ mode near 4 μm, as seen in the bottom panel of Figure 6b.

This active tunability of the FP resonance in the ENZ-FP cavity opens up exciting possibilities for dynamic, multi-level tuning of the polariton dispersions. Particularly, the multi-state switchability of GST’s phase has been shown to allow fine control over its optical response, owing to partial crystallization that leads to intermediate levels of refractive index between the crystalline and amorphous phases [52]. Assuming that the refractive index of GST varies linearly between its two phases as a function of crystallization fraction (*τ*), the mid-IR response of the ENZ-FP cavity as a function of *τ* is investigated. Figure 6c plots the wavelength dependence of *R*_*s*_ of the mid-IR ENZ-FP cavity at *θ* = 45° varying *τ* from 0 % (top) to 100 % (bottom). The plot indicates that by varying the refractive index of GST, a systematic, quasi-continuous tunability of the FP resonance condition is achieved. The thickness of GST here is set to *d* = 180 nm such that the resonances span the entire wavelength range from 3 to 5 μm as *τ* is varied. Figure 6d shows the corresponding plots of *R*_*p*_. At *τ* = 0 and 100 %, the FP resonances are far away from the ENZ mode at 4 μm, leading to two spectrally distant resonant features corresponding to the uncoupled ENZ and FP resonances. At intermediate values of *τ*, the bare FP resonance approaches *λ*_*ZE*_ resulting in distinct, *τ*-dependent, strongly coupled ENZ-FP resonances typified by the avoided crossing of dispersions around 4 μm. Dashed lines in Figure 6d highlight this avoided crossing. The multilevel resonances that can be finely controlled by the crystallization fraction leads to multiple-state dynamic switchability of the strong coupling interaction, i.e. not only can the strong coupling be switched between ‘ON’ and ‘OFF’, it can have continuously tuned states lying between these ‘ON’ and ‘OFF’ states. Such multi-level crystallization of GST may be experimentally realized using heating stages, electrical signals or optical pumping [53, 54]. Practically, studies have reported enough control over *τ* to realize over 100 distinct states, each of which correspond to different, non-volatile states of crystallization [52]. This allows one to envision applications that improve from binary to gray scale functions, especially in continuous wave-front shaping in metasurfaces and improved dynamic control of spatial light modulators [49]. Coupled with the unprecedented tunability of the polariton dispersion demonstrated here, these will be useful in the design of novel optical devices and advanced functionalities.

## 3 Conclusions

In conclusion, strong coupling of optical resonances in a planar, multi-layer system of coupled ENZ and FP cavities is demonstrated. The work exploits the unique optical properties of the interacting resonances to present a simple geometric structure where ENZ light–matter interactions can be easily tailored, with the results demonstrating an unprecedented control over the ENZ mode dispersion. The coupling strength, quantified by Ω_*R*_ and the ratio *g*, varies strongly with the thickness and field enhancement of the ENZ layer, as well as its position within the cavity. An analytical approach to elucidate these dependencies is presented by estimating field overlap of the interacting modes, which accurately predicts the trends in the variation of Ω_*R*_. The model is further validated by numerical calculations of field distribution and splitting in the system. Ω_*R*_ is shown to reach values as large as 20 % of the operating frequency, approaching the ultrastrong coupling regime, indicating unique and efficient mode coupling in a simple, planar structure. Through numerical optimization of the cavity geometry and leveraging the spectral tunability of the ENZ regime in doped CdO, the potential for extreme dispersion engineering in the system is demonstrated. Remarkably, the cavity can be designed as nearly-omnidirectional, wavelength selective polarizers, for both *s* and *p* polarizations. Analysis of the Hopfield coefficients of these cavities reveals the partial ENZ/cavity mixed nature of the generated polaritons and shows for the first time the possibility of a significant mixed mode nature far away from the crossing point of two interacting optical modes. This prediction presents a novel approach to controlling the hybrid nature of polaritons by engineering the cavity dispersion. Finally, the tunable optical properties of the ENZ layer and GST dielectric layer present the possibility of tuning the response over a wide spectral range by suitable material choice and geometry optimization as demonstrated here. In particular, the partial crystallization of GST allows quasi-continuous tuning of the cavity, providing an excellent handle for dynamic and non-volatile modulation of the SC dispersion through multiple resonant levels. The results presented here shed light on controlling and engineering ENZ light–matter interactions through coherent processes and are promising for the development of IR optical components e.g., thermal emitters [20, 27] with active and tunable functionalities.

## 4 Methods

### 4.1 Material properties

The Drude model is used to calculate the frequency-dependent permittivity *ϵ*(*ω*) of Ag and CdO, given by

(6) $$\epsilon \left(\omega \right)=\epsilon_{\infty }-\frac{\omega_{p}^{2}}{\omega^{2}+i\gamma \omega }$$

where *ϵ*_∞_ is the high-frequency permittivity, *ω*_*p*_ is the plasma frequency and *γ* is the scattering rate. The Drude model parameters for Ag are *ϵ*_∞_ = 5, *ω*_*p*_ = 8.9 eV and *γ* = 0.039 eV [55], and the fixed parameters in the Drude model for CdO are *ϵ*_∞_ = 5.3 and *γ* = 2.8 × 10^13^ rad/s [28] while *ω*_*p*_ is varied. In the low loss case (*γ* ≪ *ω*_*p*_), the real part of permittivity (*ϵ*′) becomes zero at the zero-epsilon frequency $\omega_{ZE}=\omega_{p}/\sqrt{\epsilon_{\infty }}$ . The corresponding wavelength is denoted as *λ*_*ZE*_, which lies in the UV for Ag and can be tuned in the near to mid-IR range for CdO [28]. In this work, CdO is considered throughout as the ENZ material with its *λ*_*ZE*_ as a tunable parameter.

The cavity dielectric materials used are either PMMA, having a refractive index *n*_*d*_ = 1.47 at near-IR wavelengths [56], or GST, which is a phase change material whose refractive index varies depending on its phases (amorphous or crystalline). In the mid-IR wavelength range where the properties of a GST-integrated FP cavity are analyzed, the complex refractive index varies approximately from a value of 6 in its crystalline state to 3.5 in its amorphous state, with the corresponding loss changing from ≈0.05 to practically 0 [45]. The permittivity or refractive index plots are given in Section S1 in Supplementary Material.

### 4.2 Transfer matrix calculations

A custom written transfer matrix method (TMM) code is used to calculate the reflectance and electric field distribution in the multilayers investigated here. The TMM calculations are described in Section S8 in Supplementary Material.

## Supplementary Material

Supplementary Material Details
