# Supplementary material for: Dispersion engineering of infrared epsilon-near-zero modes by strong coupling to optical cavities
Source: Nanophotonics. 2023 Jun 21;12(16):3301–12. doi: 10.1515/nanoph-2023-0215 (PMC11501747; doi:10.1515/nanoph-2023-0215)
Supplement: Supplementary file 1 — Supplementary Material Details [file j_nanoph-2023-0215_suppl_001.pdf]

Supplementary Material for

Dispersion engineering of infrared epsilon-near-zero modes by strong coupling to optical cavities

Ben Johns*

Department of Chemical Sciences, Indian Institute of Science Education and Research, Mohali, India, 140306

S1. OPTICAL CONSTANTS

Figure S1a,b plots the real and imaginary permittivity of Ag and CdO. The permittivity of CdO for three values of λ_{ZE} used in this work are shown as labeled in Figure S1b. The real (n) and imaginary (k) parts of GST refractive index in the crystalline and amorphous phases are plotted in Figure S1c,d [1].

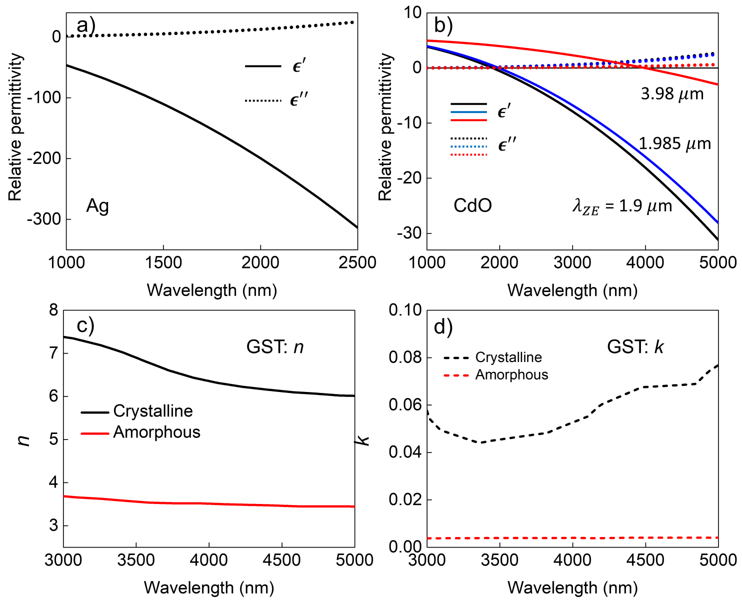

FIG. S1. (a) Permittivity of Ag, (b) permittivity of CdO with different values of λ_{ZE}, (c) real and (d) imaginary refractive index of GST in its two phases.

* benjohns@iisermohali.ac.in

S2. R_s MAPS OF ENZ-FP CAVITY

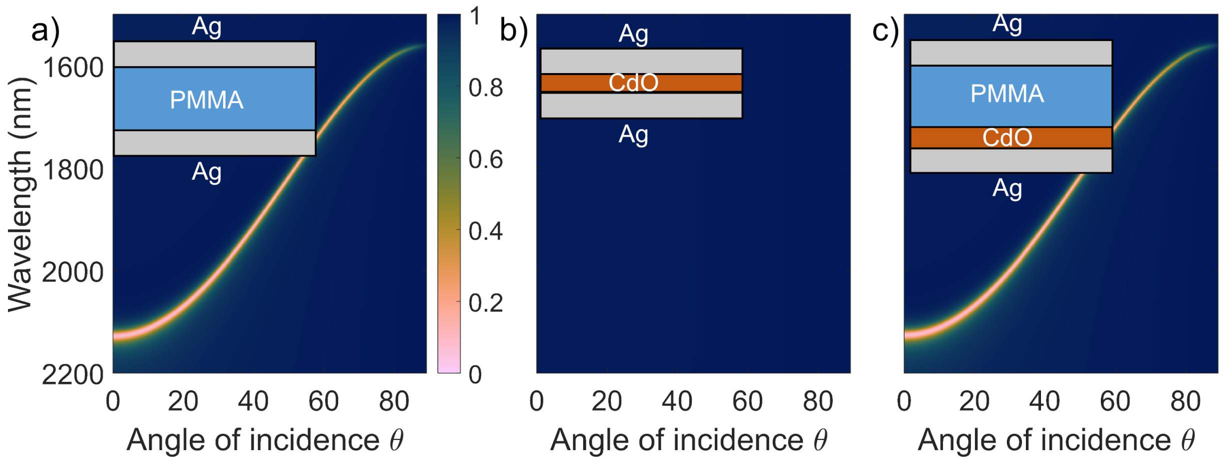

FIG. S2. R_s maps of (a) FP cavity (b) ENZ mode structure and (c) ENZ-FP cavity, corresponding to the R_p maps shown in Figure 1 in main text. All parameters are the same as in Figure 1. The absence any ENZ mode excitation in (b) implies that the ENZ-FP cavity response to s-polarized light in (c) is only due to the FP resonance.

S3. BARE DISPERSION RELATIONS

Dispersion of FP cavity

Due to imperfect reflection from the Ag mirrors, the FP cavity resonance condition is shifted slightly from that of a conventional Fabry-Perot resonator, which occurs when there is a 2π phase build-up over a round trip inside the cavity [2]. To accurately model the dispersion of the FP cavity, the condition for minimizing the reflectance of the layered structure in Figure S3a is calculated. The reflection coefficient of the four layer structure can be written as

$$r_{1234} = \frac{r_{12} + r_{234}e^{2i\delta_2}}{1 + r_{12}r_{234}e^{2i\delta_2}}; \tag{1}$$

where $r_{234} = \frac{r_{23}+r_{34}e^{2i\delta_3}}{1+r_{23}r_{34}e^{2i\delta_3}}$, r_{ij} is the Fresnel reflection coefficient for media i, j, $\delta_i = k_{zi}d$ and k_{zi} is the normal wave vector component in medium i. The phase condition to minimize

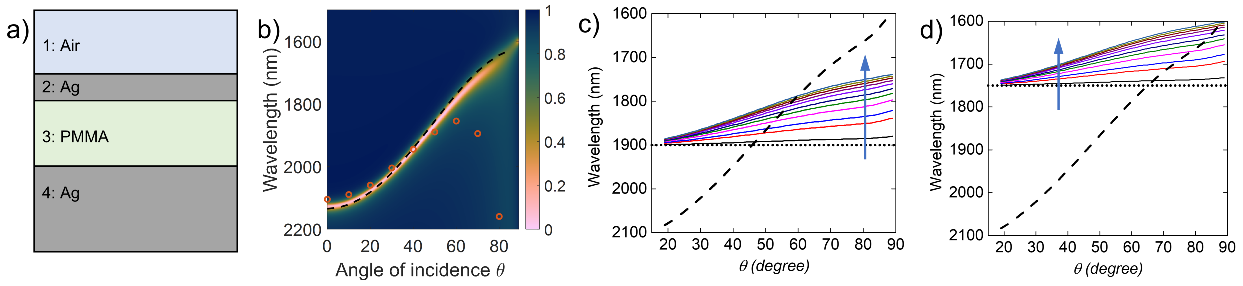

FIG. S3. (a) Schematic of 4-layer structure with air above the FP cavity. (b) Dispersion relation of FP cavity plotted as dashed black curve, overlaid on the R_p map from Figure 1a of main text. The Fabry-Perot resonance condition is identified by the red circles. (c,d) Dispersion relation of Ag-CdO-Ag ENZ mode structure for $\lambda_{ZE} = 1900$ nm and 1750 nm, respectively. d_{ENZ} is varied from 5 nm to 250 nm in each plot where the direction of increasing thickness is indicated by arrows. For comparison, the dashed curve shows the dispersion relation of FP cavity.

the numerator in Equation 1 becomes

$$\phi_{12} = \phi_{234} + 2\,Re(\delta_2) + (2m+1)\pi \tag{2}$$

where ϕ denotes the phase. Assuming low phase accumulation in the thin Ag layer i.e. $Re(\delta_2) \rightarrow 0$, the phase condition becomes

$$\phi_{12} = \phi_{234} + (2m+1)\pi \tag{3}$$

Numerical solution of Equation 3 is used to obtain the dispersion relation of the FP cavity, plotted as the dashed black curve in Figure S3b, overlaid on the reflectance map of p-polarized light. The thicknesses are $d_{top} = 20$ nm, $d = 670$ nm, corresponding to Figure 1a in the main text. The 2π phase accumulation condition also is plotted as red circles in Figure S3b, showing that at low angles, the cavity may be well approximated as a Fabry-Perot resonator. At larger angles, the deviations become more significant and the Fabry-Perot model is not well suited to describe the cavity at all angles of incidence.

Dispersion of ENZ mode

Figure S3c,d plots the dispersion relation of ENZ modes (Ag-CdO-Ag) obtained from the corresponding numerical reflectance spectra, for $\lambda_{ZE} = 1900$ and 1750 nm, respectively. The

thickness of CdO is varied from 5 nm to 250 nm with $d_{top} = 20$ nm. The blue-shifting of ENZ modes with thickness at larger angles (shown by arrows) is a well known feature that has been described in literature [3]. The FP cavity dispersion from Figure S3b is also shown here to highlight the crossing of the FP and ENZ dispersions for both the $\lambda_{ZE} = 1900$ and 1750 nm cases. The ENZ dispersions lie within ~ 100 nm range below λ_{ZE}, with the lowest thicknesses having a rather flat dispersion with $\omega_{ENZ}(k) \approx \omega_{ZE}$ (horizontal dotted lines in Figure S3c,d). The dispersion of the ENZ mode in Figure **1b** of the main text for $d_{enz} = 20$ nm is therefore approximated to be $\omega_{ENZ}(k) = \omega_{ZE}$ for the coupled dispersion calculations.

S4. ANTI-CROSSING OF DISPERSIONS AROUND THE ENZ WAVELENGTH

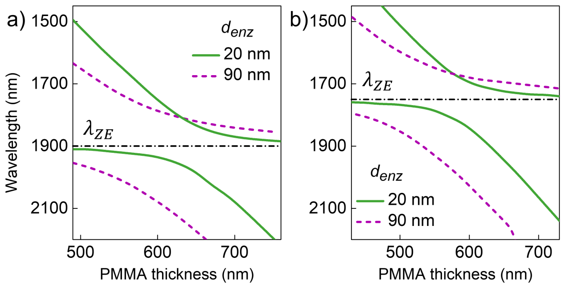

FIG. S4. Wavelength of the upper and lower branches of the ENZ-FP cavity at $\theta = 45^0$ as a function of PMMA thickness, plotted for $d_{ENZ} = 20$, 90 nm and (a) $\lambda_{ZE} = 1900$ nm and (b) λ_{ZE} = 1750 nm. Horizontal lines show λ_{ZE}, confirming that mode splitting and avoided crossing of the dispersions occur around the ENZ wavelength.

Figure S4 shows the dependence of the upper and lower branch wavelengths of the ENZ-FP cavity on PMMA thickness. The resonance wavelengths are calculated for two values of the ENZ thickness, $d_{ENZ} = 20$ nm and 90 nm at a fixed $\theta = 45^0$. Figure S4a shows the variation when the ENZ wavelength λ_{ZE} is fixed at 1900 nm and Figure S4b shows the plots for $\lambda_{ZE} = 1750$ nm. The mode splitting and anti-crossing behavior of the resonance wavelengths evident around λ_{ZE} in both Figure S4a and S4b provide clear evidence for the strongly coupled interaction of the ENZ-FP cavity around λ_{ZE}.

S5. DEPENDENCE ON ENZ THICKNESS

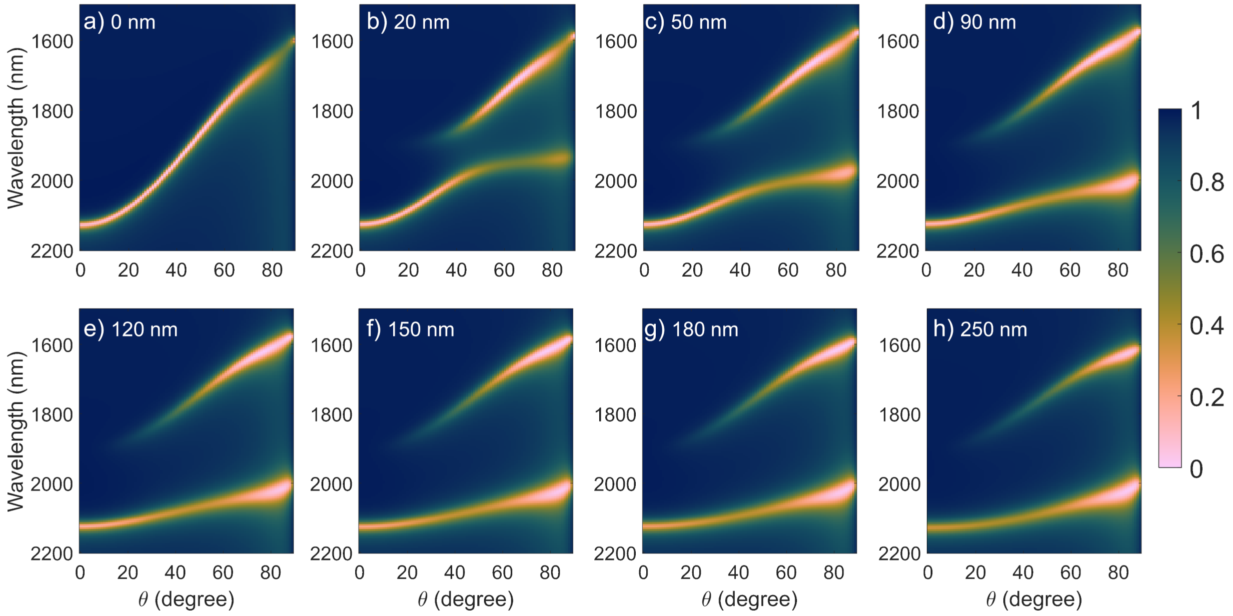

FIG. S5. R_p maps of the ENZ-FP cavity for different ENZ thicknesses from 0 - 250 nm, showing variation in dispersion splitting as a function of ENZ thickness.

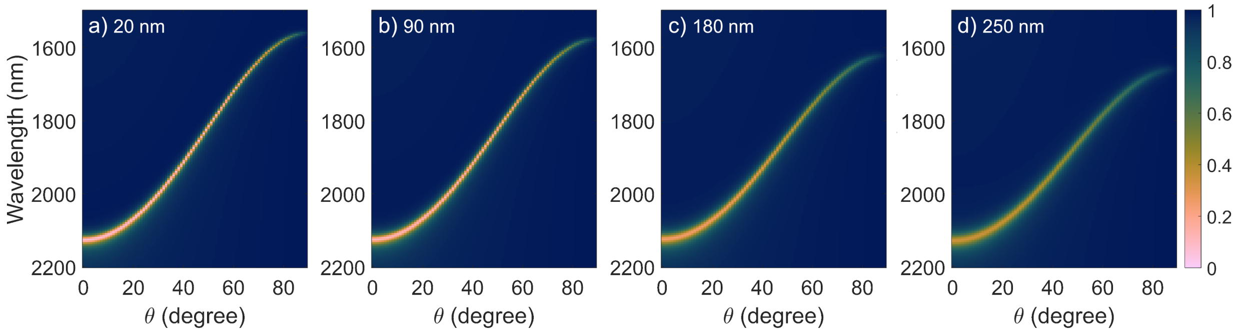

FIG. S6. R_s maps of the ENZ-FP cavity for four ENZ thicknesses and suitably adjusted dielectric thicknesses, showing that the effect of varying d_{ENZ} on the FP dispersion is offset by suitably varying the dielectric thickness.

Figure S5 shows the p-polarized reflectance maps for the ENZ-FP cavity where λ_{ZE} = 1900nm, d_{top} = 20 nm, and d_{ENZ} varies from 0 - 250 nm. It is evident that the spectral splitting increases initially and reaches a limiting value for $d_{ENZ} \sim 100$ nm, beyond which the splitting remains mostly unchanged. From a practical point of view, changing d_{ENZ} will

also affect the spectral range of the FP resonance of the ENZ-FP cavity, which is determined by the thickness and refractive index of not only the dielectric (PMMA), but also that of the ENZ layer. In order to isolate the effect of varying d_{ENZ} in the numerical calculations, any variation in the FP cavity resonance with d_{ENZ} needs to be avoided. This was done by suitably adjusting the dielectric thickness such that the FP resonance of the ENZ-FP cavity remains unmodified. This ensures that only the effect of varying ENZ thickness is observed in Figure S5. To show that the FP resonance is negligibly affected and the overall spectral range of the resonance remain unchanged, Figure S6 shows s-polarized reflectance maps of the ENZ-FP cavity for four different ENZ thicknesses. The R_s maps confirm that the FP dispersion is only slightly modified. The dispersions remain nearly identical even up to $d_{ENZ} = 250$ nm and show only slight deviations at large d_{ENZ}, especially at very large θ values.

S6. NUMERICAL OPTIMIZATION

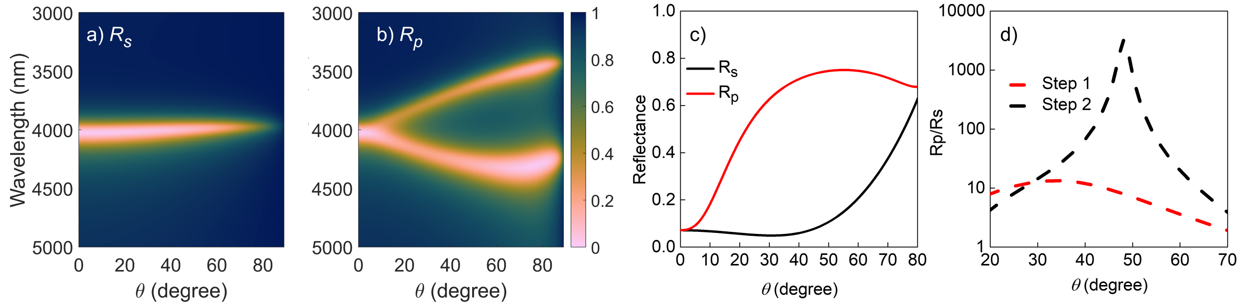

FIG. S7. (a) R_s and (b) R_p obtained after step 1 of numerical optimization in the p-polarizer scheme (see text). (c) Corresponding reflectance at the target operation wavelength $\lambda = 4000$ nm. (d) Comparison of extinction ratio after step 1 and step 2 of optimization in p-polarizer scheme.

The numerical optimization is carried out using a gradient-based constrained optimization routine in MATLAB [4]. In the s-polarizer scheme, the quantity $R_s - R_p$ at $\lambda = 2100$ nm is maximized simultaneously at angles in the range 15^0 to 70^0. This is done by defining the output of the objective function to be minimized as $-(R_s - R_p)$. The parameters simultaneously optimized are the layer thicknesses (d_{top}, d and d_{enz}) and λ_{ZE}, which are reported in the main text.

For the p-polarizer, the optimization is carried out in two steps: first, $R_p - R_s$ is maximized at $\lambda = 4$ μm by varying the four parameters mentioned above. These values are $d_{top} = 173$ nm, $d = 115$ nm, $d_{enz} = 118$ nm and $\lambda_{ZE} = 3.98$ μm. The corresponding reflectance maps are shown in Figure S7a,b. Figure S7c also plots the reflectance contrast at $\lambda = 4$ μm. While the reflectance contrast is maximized in this optimization step, it is evident that R_s is too high for efficient wide-angle polarization. To address this limitation, a second optimization step is carried out to minimize R_s where the parameters to be optimized are now only d_{top} and d i.e. d_{enz} and λ_{ZE} from step 1 are left unchanged. The results of step 2 are reported in the main text. To compare the results of the two steps, Figure S7d plots the extinction ratio (R_p/R_s) from step 1 and step 2, demonstrating a marked improvement in the figure of merit after the second optimization step.

S7. EFFECT OF DIELECTRIC REFRACTIVE INDEX ON FP DISPERSION

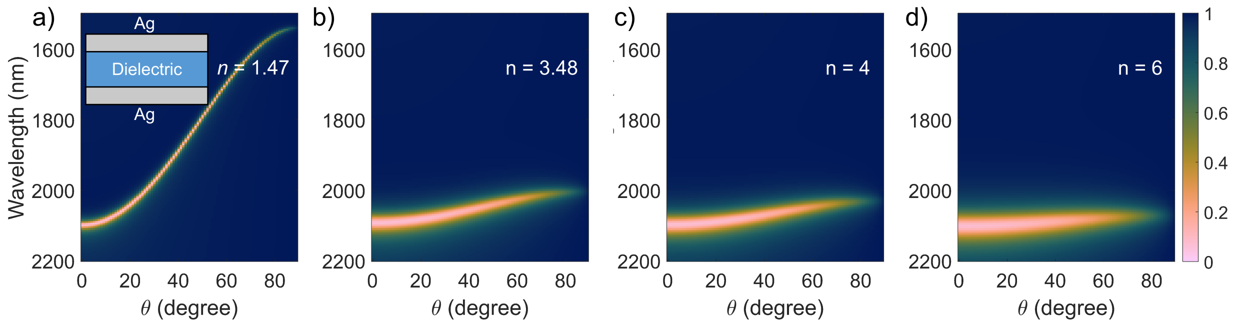

FIG. S8. R_p maps of the FP cavity shown in the inset for varying dielectric refractive indices as noted. Here, d is chosen such that the resonant wavelength at normal incidence is fixed at 2100 nm in all cases for the sake of comparison.

The angle-dependence of the FP dispersion can be mitigated to an extent by using large refractive index dielectrics, which would reduce the wavelength spread of the dispersion. Typically, Si ($n \approx 3.4$) and Ge ($n \approx 4$) are commonly used as high-index infrared dielectrics. Figure S8 plots R_s for cavity dielectric refractive index values of 1.47 (PMMA), 3.48 (Si), 4 (Ge), and 6 from left to right, showing how the dispersion becomes flatter as n increases. Even for $n = 4$, the dispersion is not flat enough for practically realizing wide-angle absorption at a given wavelength, with the FP resonance wavelength varying by ~ 100 nm in this

case. Hence GST with $n \approx 6$ is utilized to effectively achieve the wide-angle polarization described in the main text.

S8. TRANSFER MATRIX METHOD

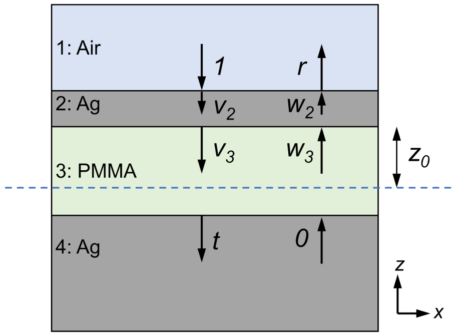

FIG. S9. Schematic of representative 4-layer structure considered below.

In planar multi-layers, the waves in each layer may be treated as having downward (-z) and upward (+z) propagating components, as shown in a representative 4-layer system in Figure S9. The stack has N media numbered 1 to N where the first and last layers are semi-infinite. v_n and w_n denote the amplitude of downward and upward moving waves respectively. In the first and last layers, these amplitudes are 1, r and t, 0 respectively, where r, t are the overall reflection and transmission coefficients of the multi-layer. v_1 amd w_1 are not defined while $v_N = t$ and $w_N = 0$. The fields in the intermediate layers can be related as,

$$v_{n+1} = v_n e^{i\delta_n} t_{n,n+1} + w_{n+1} r_{n+1,n} \tag{4a}$$

$$w_n = w_{n+1} t_{n+1,n} e^{i\delta_n} + v_n r_{n,n+1} e^{2i\delta_n} \tag{4b}$$

where r_{ij} and t_{ij} are the (polarization-dependent) Fresnel reflection and transmission coefficients for light falling from layer i to j and δ_n is the phase term in layer n. This can be written in matrix form as

$$\begin{pmatrix} v_n \\ w_n \end{pmatrix} = M_n \begin{pmatrix} v_{n+1} \\ w_{n+1} \end{pmatrix} \tag{5}$$

where $n = 2,...,N-1$ and $M_n = \begin{pmatrix} e^{-i\delta_n} & 0 \\ 0 & e^{i\delta_n} \end{pmatrix} \begin{pmatrix} 1 & r_{n,n+1} \\ r_{n,n+1} & 1 \end{pmatrix} \frac{1}{t_{n,n+1}}$. Note that M_1 and M_N are not defined since they are semi-infinite layers. By applying the above relation successively

for each layer, one obtains the matrix $\tilde{M}$ relating the waves entering and exiting the structure (called the transfer matrix),

$$\tilde{M} = \frac{1}{t_{1,2}} \begin{pmatrix} 1 & r_{1,2} \\ r_{1,2} & 1 \end{pmatrix} M_2 M_3 ... M_{N-1} \tag{6}$$

Finally, the incoming and outgoing waves are related as

$$\begin{pmatrix} 1 \\ r \end{pmatrix} = \begin{pmatrix} \tilde{M}_{11} & \tilde{M}_{12} \\ \tilde{M}_{21} & \tilde{M}_{22} \end{pmatrix} \begin{pmatrix} t \\ 0 \end{pmatrix} \tag{7}$$

which gives $t = 1/\tilde{M}_{11}$ and $r = \tilde{M}_{21}/\tilde{M}_{11}$ [5].

Having calculated $(t, 0)$, which is the same as (v_N, w_N), the fields in each intermediate layer (v_n, w_n) can be successively calculated using Equation (5). For example, to find the field along the dashed line in Figure S9 at a distance of z_0 inside the PMMA layer, we have for s-polarized light,

$$E_y(z_0) = v_3 e^{ik_{z3}z_0} + w_3 e^{-ik_{z3}z_0} \tag{8}$$

and for p-polarized light,

$$E_x(z_0) = \left[v_3 e^{ik_{z3}z_0} + w_3 e^{-ik_{z3}z_0}\right] \cos\theta_3 \tag{9a}$$

$$E_z(z_0) = \left[-v_3 e^{ik_{z3}z_0} + w_3 e^{-ik_{z3}z_0}\right] \sin\theta_3 \tag{9b}$$

The angles θ_n in layer n are calculated from Snell's law as $\sin\theta_n = \sin\theta_1/\tilde{n}$ where θ_1 is the angle of incidence at the first interface and $\tilde{n}$ is the complex refractive index of the considered layer. Further, $\cos\theta_n = \sqrt{1 - \sin^2\theta_n}$ and $k_{zn} = 2\pi\tilde{n}\cos\theta_n/\lambda$. The negative sign for v_3 in Equation (9) is due to the convention where fields traveling in opposite directions have E_z pointing in opposite directions [6].

---

[1] Ann-Katrin U Michel, Dmitry N Chigrin, Tobias WW Mass, Kathrin Schonauer, Martin Salinga, Matthias Wuttig, and Thomas Taubner. Using low-loss phase-change materials for mid-infrared antenna resonance tuning. *Nano letters*, 13(8):3470–3475, 2013.

[2] Zhongyang Li, Serkan Butun, and Koray Aydin. Large-area, lithography-free super absorbers and color filters at visible frequencies using ultrathin metallic films. *Acs Photonics*, 2(2):183–188, 2015.

[3] Nikolai Christian Passler, Ilya Razdolski, D Scott Katzer, David F Storm, Joshua D Caldwell, Martin Wolf, and Alexander Paarmann. Second harmonic generation from phononic epsilon-near-zero berreman modes in ultrathin polar crystal films. *Acs Photonics*, 6(6):1365–1371, 2019.

[4] URL `https://www.mathworks.com/help/optim/ug/fmincon.html`.

[5] Pochi Yeh. *Optical Waves in Layered Media.* John Wiley & Sons.

[6] David J. Griffiths. *Introduction to Electrodynamics.* Cambridge University Press, 4 edition, 2017. doi:10.1017/9781108333511.
